# Bat Use of Hollows in California’s Old-Growth Redwood Forests: From DNA to Ecology

**DOI:** 10.3390/ani12212950

**Published:** 2022-10-27

**Authors:** Amon J. Armstrong, Faith M. Walker, Colin J. Sobek, Cheri J. Sanville, Stephanie L. Martin, Joseph M. Szewczak

**Affiliations:** 1 Biology Department, California Polytechnic University–Humboldt, Arcata, CA 95521, USA; 2 Bat Ecology & Genetics Lab, School of Forestry, Northern Arizona University, Flagstaff, AZ 86001, USA; 3 The Pathogen and Microbiome Institute, Northern Arizona University, Flagstaff, AZ 86001, USA; 4 Australian Centre for Ancient DNA, Darling Building, North Terrace Campus, The University of Adelaide, Adelaide, SA 5005, Australia; 5 California Department of Fish and Wildlife, Eureka, CA 95501, USA; 6 North Coast Resource Management, Inc., Ukiah, CA 95489, USA

**Keywords:** bats, roosts, trees, guano, genetic

## Abstract

**Simple Summary:**

The extent of the use of tree hollows by bats is relatively unknown. However, these are vital habitats for cavern-dwelling bats to use when seeking rest, protection, hibernation, or reproduction opportunities. We collected bat guano from nearly 200 tree hollows to determine which species were present, using genetic markers, and which habitat characteristics influenced roost use. Our results indicate a different species composition than previously known in redwood trees (*Sequoia sempervirens*) on the North Coast of California. Based on the quantity of guano collected, more bats roosted in hollows with high ceilings and in forests with fewer small trees. Researchers may use the techniques of guano collection and DNA analysis presented here for the management and conservation of bat populations.

**Abstract:**

The loss of roosting resources, either through disturbance or removal, negatively affects bats. Identifying sensitive species and determining roost requirements are critical components in conserving their habitat. Cavity-roosting bats on the North Coast of California are known to use hollows in large redwood trees. In this study, we examined the factors determining the use of basal tree hollows by different bat species at eight redwood forest sites in Del Norte, Humboldt, and Mendocino Counties, California. Bat guano was collected from 179 basal hollow roosts from 2017 to 2018, and guano mass was used as an index of roosting activity. Nine bat species and one species group were identified by analysis of DNA in guano. We made a total of 253 identifications from 83 hollows into the 10 species categories. The most prevalent species were *Myotis californicus* (California myotis; 28.5% of all identifications), the *Myotis evotis-Myotis thysanodes* group (17.4%), *Corynorhinus townsendii* (17.0%), and *Myotis volans* (15.0%). We evaluated the extent to which habitat variables at the scales of the hollow, vicinity, and site influenced the level of roost use. The correlations between guano mass and habitat variables were examined using generalized additive mixed models. At the hollow scale, guano mass increased with ceiling height above the opening. At the vicinity scale, guano mass increased with less cover of small trees. At the site scale, there was no association between guano mass and distance to foraging areas, elevation, or the number of nearby hollows. These tree hollow roost preferences can inform land managers when planning the management and conservation of redwood forests.

## 1. Introduction

The cryptic behaviors of bats have led to misunderstanding by the public and limited study of their natural histories by scientists. However, the important ecological roles and vital ecosystem services that bats provide create an imperative to better understand their habitat requirements to support and conserve their populations. Bats are both prey and consumers in food webs, providing control of insect pests, which saves farmers billions of dollars annually [1]. Many bat species have adapted to developed environments by roosting in man-made structures, although they still face threats from human activities. They may be killed directly by pesticides or wind turbines, or suffer the indirect effects of human activity, such as the spread of the fungal disease white-nose syndrome, or the cascading effects of climate change [2,3,4,5]. As with most wildlife, bat populations have suffered from habitat loss due to development and resource extraction. Specifically, populations have declined through disturbance or removal of roosting resources used by maternity colonies in summer and overwintering bats in hibernacula (winter refugia) [6,7,8]. These human-caused reductions in roost availability likely limit the distribution and carrying capacity of bat populations [9,10].

On the California North Coast (Del Norte, Humboldt, and Mendocino Counties), where caves are rare, and structures for roosting are limited, bats have been documented roosting in basal hollows of trees, as in other parts of the world [11,12,13,14,15,16,17,18]. The oldest coastal redwood (*Sequoia sempervirens*) trees provide most of the basal hollow roosts, as they have endured centuries of fire scars and healing that created cave-like hollows [19]. On a landscape where 95% of the redwood forests are managed for timber production [20,21], deliberate conservation of roost trees would likely help to preserve bat populations. Quantifying the physical characteristics of hollows that bats use [22] and their surrounding environmental features [18] would enable the selective conservation of these trees. The characteristics of tree-hollow roosts are typically measured at three scales: the individual tree, the immediate vicinity, and the surrounding site or landscape e.g., [23].

Quantifying bat usage of redwood hollows presents a challenge, given how infrequently individual bats or colonies are encountered in these structures [24]. Methods such as capture, audio recording, radio-tracking, and thermal videography are labor intensive and provide limited data on bats using hollows as roosts [12,13,25,26,27]. Collecting guano, and measuring its mass, is a non-invasive alternative that provides an index of the amount of bat use, which can be used to determine preferred characteristics of tree-hollow roosts.

Guano also provides DNA, which may be analyzed for species identification. Genetic analysis of mixed guano samples is effective at identifying over 90% of species tested in the order Chiroptera [28]. Guano DNA from redwood hollows has previously been analyzed for species identification from a relatively small number of selected fecal pellets (n = 217) from two sites in Del Norte and Mendocino Counties [16,20]. Using genetic identification is an effective method to increase knowledge of species prevalence across all roosts (measured by the percentage of identifications; also known as species composition, commonness, occurrence, or frequency). With increased sample sizes, the prevalence of species of conservation concern may be evaluated for habitat needs.

Our objectives centered on species identifications by genetic analysis of guano to elucidate natural history patterns and habitat associations of bats using basal hollows on California’s North Coast. Specifically, we aimed to: (1) determine species proportions across roosts; (2) evaluate changes to species composition over a one-year period; and (3) assess habitat variables in relation to guano quantity at the scales of the tree hollow, the immediate vicinity, and the surrounding site. We evaluated eight sites from this large and diverse geographic area, collecting guano for one year from nearly 200 basal hollows. The findings of this investigation illustrate that the DNA and quantity of bat guano at roosts can provide species prevalence information and also illuminate the characteristics of redwoods that are important to bats.

## 2. Materials and Methods

### 2.1. Ethics Statement and Permits

All bats encountered were minimally disturbed in accordance with the guidelines of the California Polytechnic University-Humboldt Institutional Animal Care and Use Committee (protocol no. 15/16.B.119-A).

### 2.2. Study Site and Tree Hollow Selection

**Figure 1:**
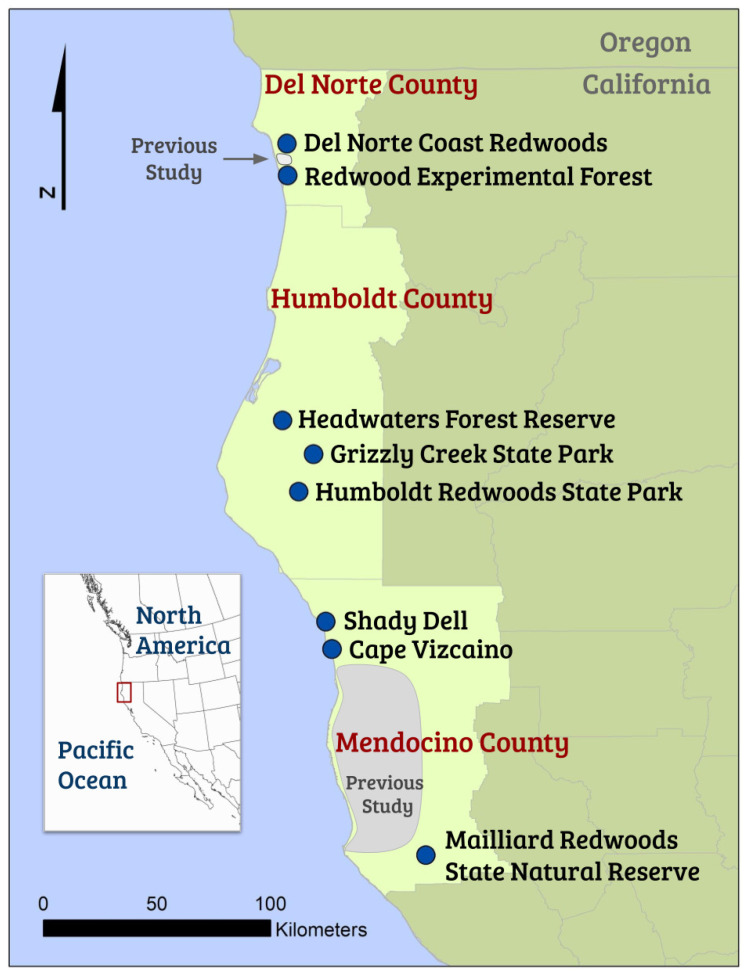
Basal hollow study sites on the North Coast of California (N-arrow denotes North; WGS 1984 UTM Zone 10 North; County boundary source: ESRI). Previous studies include Gellman and Zielinski [11], Zielinski and Gellman [12], Purdy [13], Zielinski, et al. [16], and Roberts [17].

We selected eight study sites on the North Coast of California within the redwood region spanning Del Norte, Humboldt, and Mendocino Counties (Figure 1). Old-growth forests were chosen based on their likelihood to contain basal tree hollows, which can take centuries to form [19]. Coast redwood is the main tree species, with Douglas-fir (*Pseudotsuga menziesii*), tanoak (*Notholithocarpus densiflorus*), red alder (*Alnus rubra*), and bay laurel (*Umbellularia californica*) also occurring. The understory is dominated by sword fern (*Polystichum munitum*) and huckleberry (*Vaccinium ovatum*) [21]. Twelve bat species occur in this area, including one species listed as Endangered by the International Union for Conservation of Nature (IUCN), the little brown bat (*Myotis lucifugus*), and two Species of Conservation Concern in the State of California: the Townsend’s big-eared bat (*Corynorhinus townsendii*), and pallid bat (*Antrozous pallidus*). Other bat species present in this area include the big brown bat (*Eptesicus fuscus*), fringed myotis (*M. thysanodes*), long-eared myotis (*M. evotis*), long-legged myotis (*M. volans*), Yuma myotis (*M. yumanensis*), California myotis (*M. californicus*), Mexican free-tailed bat (*Tadarida brasiliensis*), silver-haired bat (*Lasionycteris noctivagans*), and hoary bat (*Lasiurus cinereus*) [11].

**Figure 2:**
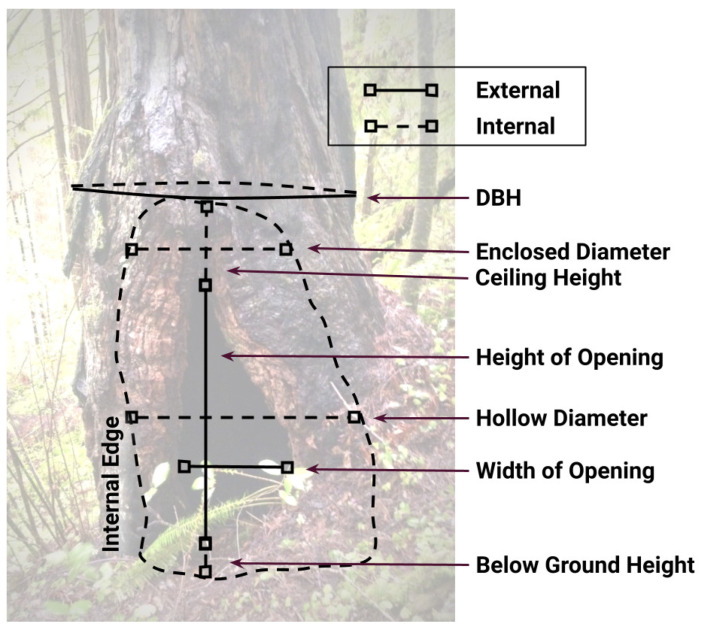
Diagram of basal hollow measurements (meters). DBH = Diameter at Breast Height; Enclosed Diameter = internal diameter above opening; Ceiling Height = height from top of opening to ceiling; Below Ground Height = height from ground level to bottom of hollow.

At each site, we located tree hollows with initial guidance from the participating land managers. We searched forests of various ages within sites, but basal hollows were mainly found in old-growth forests, apart from a few retained “legacy” trees. From the initial hollows identified, we searched forests within a radius of at least 100 m from each new hollow to locate other hollows (see Supplementary Figure S1). Half of the searches were intentionally conducted off roads or trails to reduce anthropocentric bias. We recorded all hollow locations with a Global Positioning System (GPS) and mapped them with ArcMap 10.4.1 (ESRI, Redlands, CA; WGS 1984 Zone 10 North, Figure 1). We included basal hollows if they met the minimum threshold of having closed tops with ceilings above the cavity opening [11]. There was no minimum size of the hollow opening. Hollow dimension measurements were based on field tests and established methods (Figure 2, Table 1) [11,16].

### 2.3. Guano Sampling

To collect guano, we stapled a water-permeable screen (3M Weedblock, 3M Company, St. Paul, MN, USA) inside of each hollow near the substrate to prevent bat interactions with the screen. To achieve a robust measure of use per hollow by bats, we collected guano monthly for at least one year per site (overall study period: April 2017–September 2018). A weighted interpolation of guano mass was used to equalize monthly comparisons when guano collections extended beyond one month after the previous collection or when the traps were disrupted by bears, people, or logging operations. We stored the guano in a freezer at −5 °C and removed it only once to avoid DNA degradation.

We separated guano pellets from tree debris and other detritus, recorded the mass (g), and placed the samples in a DNA stabilizer (RNAlater, Life Technologies, Carlsbad, CA, USA). The guano was not oven-dried to standardize mass because the DNA would likely degrade. Most guano was dry when collected and dried more in paper envelopes prior to processing (samples were stored in a freezer for up to 6 months). To determine which bat species roosted in each hollow, we performed DNA metabarcoding analysis on 236 guano samples at the Northern Arizona University (NAU) Species from Feces lab. This included 169 samples pooled per hollow from one year of collections, 10 from half-year collections, and 57 from monthly collections (to examine species composition change over time; presented in figures and tables using Google Workspace).

### 2.4. DNA Metabarcoding for Bat Species Identification

We analyzed the pooled guano samples, rather than individually selected pellets [16], to increase the genetic information per roost [29]. We successfully extracted the genomic DNA and amplified a short section of cytochrome oxidase subunit I (COI) from the samples using our standard methodology [28,29]. The amplified product was sequenced on an Illumina MiSeq V3 600 cycle kit (Illumina, Inc., San Diego, CA, USA) to obtain DNA sequences of one or more taxa per sample. We computationally processed sequencing reads using QIIME2 v2020.11 [30] to obtain read variants of the highest taxonomic quality. Priming regions were removed using cutadapt v3.1 [31] to isolate the 202 base pair fragment of interest. We removed the low-quality reads, alleviated sequencing contamination by joining paired-end reads, and filtered out PCR artifacts (chimeric reads) using DADA2 [32]. Using our positive control containing a known mixture of nine bat species of three families, we identified a read threshold by which to filter out read variants of likely sequencing error. Sequences were then classified using a naïve-Bayes machine learning classifier [33] that we trained against our custom reference database. We retained species classifications only if they were classified with at least 90% bootstrap support. Any read variants not classified using the machine learning algorithm to species were cross-referenced against the National Center for Biotechnology Information’s (NCBI) GenBank database [34] using BLAST [35] with taxa classified using Least Common Ancestor (LCA) analysis in MEGAN v6 [36]. This cross-referencing step helped to alleviate any false negative bat classifications in the naïve-Bayes model or identification of non-bat taxa.

### 2.5. Vicinity Vegetation and Site Variables

We estimated the proportion of vegetation cover (upper canopy, lower canopy, shrub, and herbaceous) within a 10 m radius around each tree following the CDFW-CNPS Protocol for the Combined Vegetation Rapid Assessment and Relevé Field Form (CNPS.org; updated 5 June 2019). To measure the density and size class of trees at the vicinity scale, we counted trees and recorded diameter categories (< or >60 cm DBH) within a 30 m radius.

For site-scale predictor variables, we recorded the elevation and distances between hollows and streams, roads, and clearings to use in determining potential effects on roost selection. Geographic Information System (GIS) layers were accessed for streams, roads (polylines; USGS, Caltrans), and clearings (vegetation rasters; National Agriculture Imagery Program [NAIP] aerial imagery 2012; CDFW Map Services). The number of nearby hollows was determined using GPS locations and the “Near” tool in ArcMap.

### 2.6. Mixed-Effects Models

We used an information-theoretic approach to evaluate the influence of habitat (predictor) variables on the mass of bat guano (response variable) at three scales: hollow, vicinity, and site [37]. In all analyses, we considered the hollow to be the experimental unit. The index of guano mass was related to the number of bats but was not a true count because the rate of guano deposit was unknown. Similarly, species detections from DNA analysis are not necessarily proportional to the amount of use by those species due to biases in the process of sampling and iterative testing.

We evaluated Generalized Additive Mixed Models (GAMMs) using the “mgcv” package in program R [38]. GAMMs incorporate fixed effects and random effects to resolve data autocorrelation in certain variables. In our analysis, the fixed effects were the habitat variables, and the random effect was the “site” variable (n = 8), chosen due to spatial autocorrelation. The GAMM general form is as follows:

(1) $$g{\left(E\left(Y\right)\right)}_{ij}=\beta_{0}+f_{1}\left(x_{1}\right)+f_{2}\left(x_{2}\right)+\ldots +f_{i}\left(x_{j}\right)\;\sim \;N\left(0;\;\sigma^{2}\right)$$

where *g* is a smoothed function of *E*, the expected quantity of *Y*, the response variable (guano mass), β is the intercept, and *f* is a smoothed function of *x*, the predictor variable from the *j*th collection at hollow *i*, and *i* = 1…131, and the random intercept (site), which is normally distributed (*N*) with mean 0 and variance σ^2^. The models were cross-validated using leave-one-out training and testing portions of the full dataset. To rank the models, we used Akaike’s Information Criterion corrected for low ratios of the sample size to the number of estimated parameters (AICc) [37]. Top-ranked models provided evidence for characteristics of a roost most likely to be important for bats.

## 3. Results

### 3.1. Guano Collection and Evidence of Maternity Colonies

The monthly guano collections revealed consistent depositions at 139 hollows for at least one year. We searched secondary (previously logged) forests within several sites, but the fire scars were not deep enough to install guano traps, as hollow formations can take hundreds of years. The hollows were primarily in redwood trees (n = 130). Guano was collected from nearly every hollow (132/139) at least once over the study period. The total mass of the guano collected was 1014 g (~100,000 pellets) over 1547 visits to hollows during the study period (mean = 1.07 g/sample, SE = 0.24; 925 samples obtained; 622 visits without guano; Table 2).

**Figure 3:**
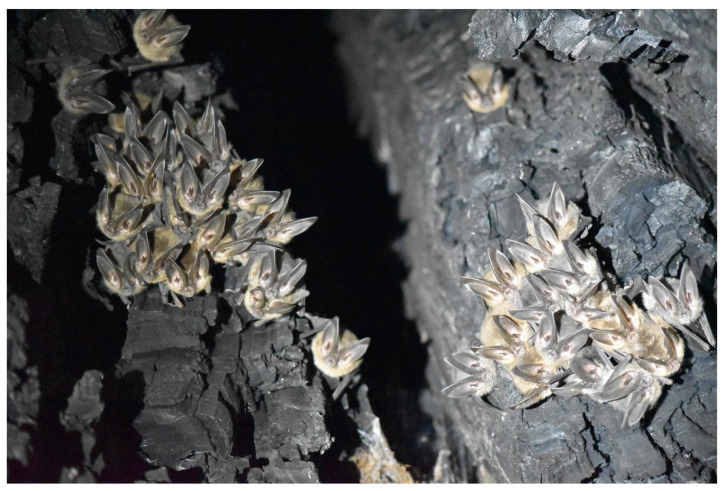
A maternal colony of approximately 40 *Corynorhinus townsendii* on 31 August 2017 in hollow 18 at Grizzly Creek State Park, Humboldt County, California. Photograph: A. Armstrong.

Observations of bats were rare, with only 13 individual or colony observations in 1547 hollow visits. Accurate visual and photographic identifications were possible in some cases, such as the colony of approximately 40 *C. townsendii* at Grizzly Creek 18 in August 2017 (Figure 3). The presence of identified species was confirmed by genetic analysis of pooled samples in some cases, although guano could not be matched to specific bats. Some identifications based on guano were also countered by genetic analysis. For example, the colony at Mailliard Redwoods 08 in the summer of 2017 was presumed to be *C. townsendii* based on the large size and golden color of guano pellets; however, the species identified in DNA sequences were *M. volans*, *M. evotis/thysanodes* and *M. californicus* (from the first 6-month sample). In the second six-month sample, all sequences identified *A. pallidus*, which was unknown to roost/breed in the region’s tree hollows.

### 3.2. Bat Species Detected via DNA Metabarcoding

**Figure 4:**
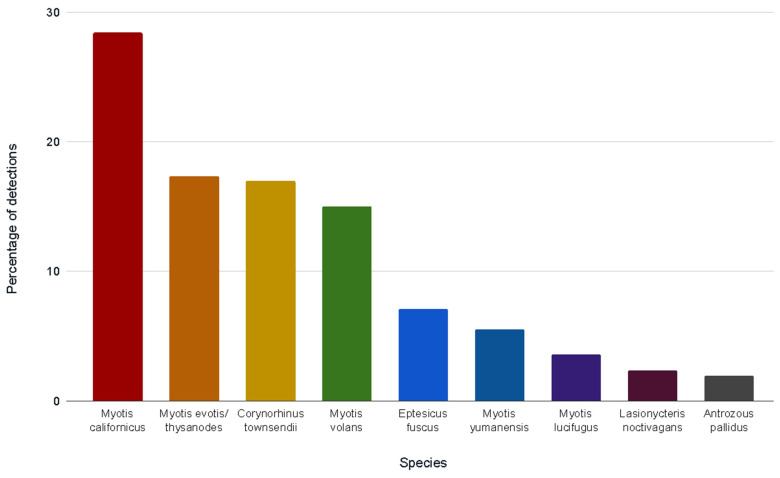
Bat species identifications, by percentage of total species detections (n = 253; multiple species per tree), from DNA analyses of guano collected from 83 tree hollows during 2017–2018 in Del Norte, Humboldt, and Mendocino counties, California (1 year; 8 sites). *Lasiurus cinereus* (absent in figure) was detected once (0.4%) at the Shady Dell site. See Table 2 for species-by-site detections.

Our positive control amplified and sequenced correctly, and no negative controls amplified. Bat species were successfully identified in 121 of 236 samples submitted for DNA analysis, pooled by hollow. Of 98 single pellet samples, the DNA in 55 pellets was successfully amplified to return species identification. In most cases, multiple species were identified using each hollow over the study period. Nine bat species and one species group were identified in 253 identifications from 83 hollows (Figure 4).

The most prevalent species, by the proportion of DNA identifications, was *M. californicus* (28.5% of all identifications; 72/83 hollows). Although this species cannot be separated from *M. ciliolabrum* (western small-footed bat) by DNA analysis, capture records do not indicate that California’s North Coast is part of the range of *M. ciliolabrum*. The *M. evotis-M. thysanodes* group was identified as the second most prevalent (17.4%; 46/83 hollows; species indistinguishable by DNA analysis). *C. townsendii* followed closely as the third most prevalent species (17.0%; 43/83 hollows; Figure 4). The number of species identifications per site provided a more specific picture of which sites contributed to the proportions in Figure 4 (see Table 2).

### 3.3. Species Composition Change over Time

**Figure 5:**
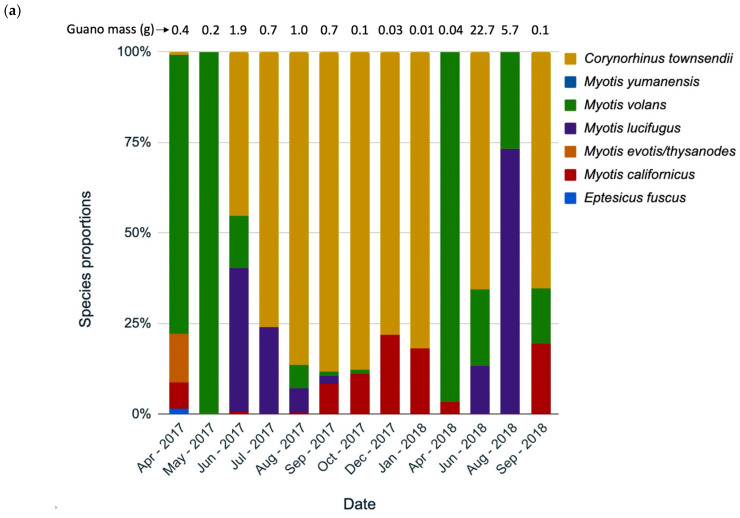

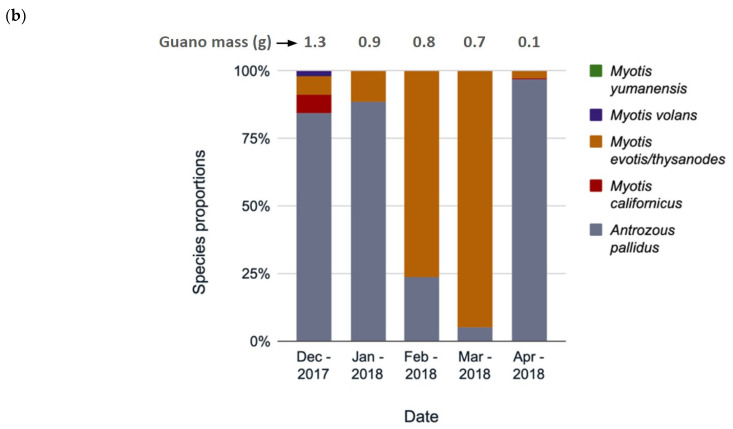
Species identifications by percentage of monthly guano collections at hollows (**a**) Grizzly Creek 22, and (**b**) Mailliard Redwoods 01, California. Some months are missing due to unsuccessful amplification of DNA.

Species composition changed throughout the year, according to the guano collections from three hollows analyzed separately by collection date (Grizzly Creek 22, Mailliard Redwoods 01 [Figure 5a,b], and Grizzly Creek 01). Although we had a limited sample size, data on species composition change in roosts are extremely sparse, so we chose to present two hollows here as natural history observations. In Grizzly Creek 22, at least seven species roosted from April 2017 to September 2018, usually with several species in the same month (Figure 5a). *C. townsendii* had the highest prevalence in most months, except May 2017 and April in both years, when *M. volans* was most common (Figure 5a). *M. lucifugus* was detected more in summer months, and *M. californicus* was detected more in winter months (indicating possible hibernation). In Mailliard 01, five species were detected, with *A. pallidus* present in all months and *C. townsendii* absent. *M. evotis/thysanodes* were detected in all months. These species composition results revealed patterns worthy of further investigation.

### 3.4. Multimodel Inference of Tree Hollow Characteristics Important to Bats

GAMM models were run to determine which hollow characteristics influenced the total amount of guano deposited. From 31 candidate models run, the weight of evidence by AICc value was not strongly in favor of one top model (Table 3). Since this weight of evidence for the top model was weak, inference about the effects of these combined variables may be based on the top several models with the highest weights (multimodel inference; Table 3).

Ceiling height was included in every model and had the strongest effect of any variable (β = 0.19, SE = 0.045), indicating its influence on the mass of guano deposited, hence the amount of bat use (see Supplementary Figure S2). In multivariate models, other variables with some influence on bat use were the maximum height and maximum width of the hollow opening, the volume of the hollow cavity, and DBH. The models were based on a dataset with five hollows removed due to extreme guano masses or extreme hollow measurements. For example, the data for the maximum height of the hollow opening were skewed by the strong influence of Mailliard Redwoods 08, which had the highest opening and mean guano mass by far (15.5 m and 23.0 g, respectively), but when removed from the analysis, maximum height became a much less significant variable. Conversely, outlier hollows Experimental Forest 02 and Del Norte 15 had extreme measurements for maximum diameter of the enclosed hollow and maximum height, but very little guano was collected at the two sites.

### 3.5. Model Inference of Vicinity and Site Characteristics Important to Bats

**Figure 6:**
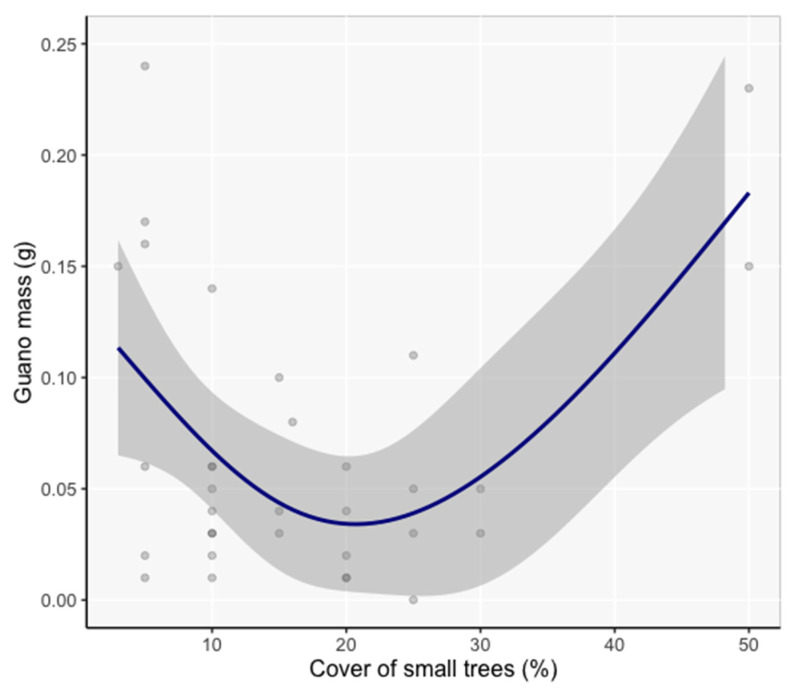
Generalized Additive Mixed Model spline function of guano mass in response to small tree cover (family = Gaussian; link function = identity, n = 36). Shaded areas are 95% confidence intervals.

At the vicinity scale, the cover of small trees was the only variable with significant influence on guano mass (β = −0.17, SE = 0.057, *Z* = 2.97, P = 0.003, Figure 6). The frequentist statistics are reported here because the evidence ratio for the next best model was 1:1, indicating that adding other vegetation variables to the model did not improve it.

At the site scale, GAMMs were used to assess the effects on the guano mass of distance to open foraging areas, elevation, and the number of nearby hollows. Over all sites, no models with single variables or variable combinations performed better than the null model, indicating a lack of significant effect.

## 4. Discussion

Basal hollows in old-growth redwood forests on California’s North Coast provided roost sites for at least ten bat species over our study period in a region that lacks other cave-like roosting features. Basal hollows could not be found in secondary (previously logged) forests, making old-growth forests particularly important roosting habitats for bats. The methods of guano collection and genetic analysis used in this study have been proven to effectively determine important roost habitat characteristics and species use by bats. Species identifications by sight were rare (>1% of identifications), and acoustic recording and capture at tree roosts were previously found to be ineffective [12,13,20,39,40]. The *M. californicus* and the *M. evotis–M. thysanodes* groups were identified in hollows at all sites, indicating the importance of this roost type for these species. *C. townsendii* was identified in seven out of eight sites, which greatly expanded knowledge of their relative abundance in basal hollows. Previously, this species was rarely encountered on the North Coast and was a candidate for listing as threatened by the State of California. This investigation also identified maternal colonies and likely hibernacula, which are essential to the persistence of bat populations. Our study provided further evidence of the value of basal hollow tree roosts for bats and, thus, the need to conserve this resource in forests worldwide.

### 4.1. Genetic Species Identification

Bat species were identified more completely with the newer “Species from Feces” DNA mini-barcode assay [28] than by Mazurek and Zielinski [20] and Zielinski et al. [16]. Previously, members of one group (*M. evotis*-*M. lucifugus carissima*-*M. thysanodes*) could not be distinguished from each other [41]. DNA analysis at the NAU lab successfully isolated *M. lucifugus* but was limited to the pair groupings of *M. californicus–M. ciliolabrum* and *M. evotis–M. thysanodes*. Although *M. californicus* and *M. ciliolabrum* are not different in the sequence divergence of mitochondrial DNA, range records indicate that all identifications were *M. californicus* [10,12,13,42]. Both species in the *M. evotis–M. thysanodes* group have been identified inside hollows in Del Norte County after being captured [13].

The most common DNA species identifications from guano were different from previous basal hollow studies on the North Coast. Most prominently, the percentage of *C. townsendii* identified using redwood trees was much higher than in previous guano-based studies (17.0%; versus 1.3% in Zielinski et al. [16], Figure 4). Further, acoustic monitoring by Kennedy et al. [40] in Humboldt Redwoods State Park identified *C. townsendii* in only 0.24% of total detections. The most common species in our study, *M. californicus* (28.5% of all identifications), was also different from the results of Zielinski et al. [16] and Mazurek and Zielinski [20], who recorded *M. volans* as the most common species using basal hollows (35.6% and 46% of pellets analyzed, respectively). The *M. californicus-M. ciliolabrum* group also ranked high in those studies, with a 28.2% proportion of pellets and inhabiting 73% of hollow-bearing trees [20]. We identified three novel species using basal hollows, all in Mendocino County: *Lasionycteris noctivagans* (2.4% of detections), *A. pallidus* (2.0%), and *L. cinereus* (0.4%; one detection). Other than *A. pallidus*, these species and *Tadarida brasiliensis* are typically recorded flying above the redwood canopy [40].

While the DNA mini-barcode assay is sensitive and able to identify species from one pellet in a sample of 100 pellets, environmental degradation may have reduced the success of DNA amplification (needed for sequencing) in the guano samples. The lack of sequencing success in about half of the guano samples was likely a consequence of environmental stressors such as moisture, ultraviolet light, and warm temperatures prior to collection [29]. Nearly 100% of guano samples can be sequenced successfully when stored for up to 30 months in dry, dark, and cool conditions [29]. Conversely, nearly all (16/17) tests of samples from caves with high humidity and cool temperatures were unsuccessful after 12 months of storage [29]. Similar conditions could have reduced success in our study, as coastal redwood forests tend to be foggy and humid. More frequent collection and preservation may have increased DNA identifications.

### 4.2. Species Use by Month

Changes in species composition were revealed in the three hollows with monthly species identifications at the Grizzly Creek and Mailliard Redwoods sites. Continuously high proportions of *C. townsendii* in Grizzly Creek 22 over winter suggested possible hibernation and added to limited knowledge of winter distribution for this species. Likewise, the high proportion of *A. pallidus* in winter in Mailliard Redwoods 01 indicated a likely hibernaculum. In months when multiple species were detected, species mixing (using the same roost at the same time) could have occurred, but this was not confirmed and has rarely been observed or studied. Species mixing was only observed “occasionally” in a long-term study of Vespertilionid bats in Europe from 1968 to 2007 [43]. At another European location, bats in the same roost hollow separated themselves by species, with minimum interaction [44]. Currently, knowledge of inter- and intra-specific social interactions is important to monitor for potential transfer of disease, especially white-nose syndrome. While white-nose syndrome is mainly found in cave-dwelling bats, large tree-hollow roosts are analogous to caves and are likely places of interaction.

### 4.3. Variables Affecting Hollow Use

#### 4.3.1. Hollow Scale

Guano mass was higher in large basal hollows, which corroborated previous studies indicating a preference by bats for high-volume roosts [12,13,20]. The top predictor for bat use of basal hollows was the height of the ceiling above the opening. For example, the hollow with the most guano in one collection (Mailliard Redwoods 08; 130 g) also had the tallest opening and highest ceiling. Individual bats, and especially maternal colonies, in such tall internal spaces, may be seeking thermal regulation, as higher ceiling hollows were protected from weather and tend to maintain consistent temperatures [14]. Higher ceilings may also have decreased the ability of ground-based predators to access the roosting bats [23]. Higher hollow volumes have been posited as better for predator avoidance because of the ability of bats to escape with more flight area [12]. However, volume, by itself, was not significantly correlated with roost use by bats (β = −0.12, SE = 0.08), but volume was influential when included in models with other top variables. Our result was different from previous local studies in which hollow volume was significant alone and in models with other variables [11,13,17].

The maximum width of the hollow opening was also an important characteristic in roost selection, possibly because bats in flight could maneuver into hollows more easily with wide openings. Nevertheless, guano was collected from several hollows with relatively small openings, which bats possibly selected to avoid predators [45]. If bats actually “selected” roost trees from several options, it follows that external tree diameter (DBH) may have been a cue for hollow size, as it was correlated with internal hollow diameter (*r* = 0.66). DBH did not increase roost use (guano mass) in univariate regression models, which corroborated Gellman and Zielinski [11], although DBH was positively correlated to roosting activity in other locations [22,46,47].

#### 4.3.2. Vicinity Scale

At the vicinity scale (10 m radius), roost use increased in hollows with a smaller proportion of cover in the lower canopy (small, young trees). A preference to roost in forests with less cluttered low-to-middle canopy space was most likely related to easier navigation, roost relocation, and increased warmth from sunlight to benefit developing young [48]. For tree-roosting bats worldwide, increased roosting has been associated with lower canopy cover [48]. Slow-flying bats, such as *C. townsendii* and *Myotis* species that have low body mass and low wing-loading to promote maneuverability [49], are more capable of navigating understory vegetation to access basal hollows in larger trees. As human-created edges increase, from timber cuts, road incursions, and other developments, lower canopy tree species compete for space and resources [50] and reduce roost accessibility. Our finding provides supporting evidence that forest edges influence species composition, in this case by restricting less maneuverable bats from forest interiors.

#### 4.3.3. Site Scale

At the site scale, we did not find that hollow use was associated with shorter distances to foraging areas, such as clearings, roads, or streams. Bats have been hypothesized to roost more frequently near forest edges to reduce the energy used flying between roosting and foraging habitats e.g., [26]. Particularly, we expected to find an association between roost use and distance to streams based on the results of previous studies e.g., [11,51]. While bats often forage on clearing and road edges, their use of streams is higher due to the increased density of insect prey [52] and the availability of drinking water [53]. However, congruent with our study, there has not always been a positive correlation between roosts and the distance to water e.g., [53,54]. A confounding factor may be that ephemeral water sources unavailable in spatial data layers may be used for drinking. Additionally, hollows near foraging areas were likely used as feeding roosts, as evidenced by found moth wings, increasing guano deposition [55].

For site variables, our use of mixed-effects models (GAMMs) to avoid issues of spatial autocorrelation by site tempered the magnitude of effects. This is a consequence of attempting to generalize environmental effects on roost selection across a wide geographic range. Models run without consideration of site autocorrelation (the random effect in mixed models) resulted in stronger correlations between guano mass and both distance to streams and hollow volume over all sites. We suggest that in future studies, regression analyses should be stratified by site or split into high and low guano-producing hollows in order to parse out the site-specific needs of bats.

## 5. Conclusions

We found that redwood basal hollows were used by two Species of Special Concern in the State of California and one Endangered species (*M. lucifugus*; IUCN, US Fish & Wildlife Service “Under Review”). Basal hollows provide an important roosting resource to these species and other bats, as they provide respite, protection, and conditions for reproduction. The availability of hollows likely limits the distribution and abundance of bats in forests globally [10]. The use of basal tree hollows may alleviate the stress of roost scarcity in areas where caves and mines are rare. The importance of tree hollows to wildlife has been quantified and described on the North Coast and in forests worldwide e.g., [56].

Bat monitoring at roosts can be improved by including guano collection and DNA analysis as effective methods for researchers and forest managers to quantify and identify bat species use. The continuing reduction in costs of DNA analysis will make more information accessible to a broader range of researchers. Field or lab testing kits that could be used with modest expense and expertise would quickly increase bat species data. DNA from guano can provide data beyond bat identification, such as sex ratios, dietary preferences, and identification of other animal species using hollows. Validating these methods will require additional testing of DNA deterioration times under different conditions. Combining DNA analysis with other research methods will provide more detailed information about the effects of roosting in particular hollows on the fitness of individual bats, colonies, and local populations. This level of knowledge is needed for proper habitat management and to guide mitigation as natural roost locations are altered [57]. Continued research on the ecological intricacies that make tree hollows suitable for bat use will lead to forest management practices that conserve the best habitat for the most species.

## Figures and Tables

**Table 1:** Tree-hollow characteristics used as predictor (independent) variables for bat use, as indexed by guano mass. All measurements in meters; vegetation cover estimated by percent.

Hollow	Vicinity	Site
Diameter at Breast Height (DBH)	Cover	Distance to water
Aspect of opening	Canopy	Distance to road
Max. height of opening	Upper (tall trees)	Distance to clearing
Max. width of opening	Mid (small trees)	Elevation
Max. diameter-internal	Shrub	Number of hollows
Ceiling height above opening	Herbaceous	within 300 m
Max. diameter above opening	Within 30 m radius:	
Volume ($\pi r^{2}h/3)$	Tree species	
Vegetation covering opening	Tree DBH (< or > 60 cm)	
	Tree density	

**Table 2:** Bat prevalence (species frequency) by percentage per site, based on species identifications from DNA analysis of pooled guano collections from 83 hollows over the study period (April 2017–September 2018), at eight sites on the North Coast of California. Asterisks indicate: * Species of Conservation Concern in the state of California; ** Species listed as Endangered by the IUCN.

	Del Norte Redwoods	Experimental Forest	Headwaters Forest	Grizzly Creek	Humboldt Redwoods	Shady Dell	Cape Vizcaino	Mailliard Redwoods
Hollows per site	15	10	24	25	26	13	12	9
Number of species	3	7	58	40	52	33	28	32
Species percentage per site								
*Myotis californicus*	33.3	42.9	29.3	17.5	34.6	21.2	42.9	21.9
*Myotis evotis/thysanodes*	33.3	28.6	13.8	20.0	25.0	9.1	3.6	31.3
Corynorhinus townsendii *		14.3	15.5	20.0	11.5	27.3	25.0	9.4
*Myotis volans*	33.3		22.4	17.5	13.5	12.1	3.6	18.8
*Eptesicus fuscus*		14.3	8.6	5.0	7.7	6.1	14.3	
*Myotis yumanensis*			10.3	10.0		9.1		3.1
Myotis lucifugus **				10.0		15.2		
*Lasionycteris noctivagans*					7.7		7.1	
Antrozous pallidus *								15.6
*Lasiurus cinereus*							3.6	

**Table 3:** Generalized Additive Mixed Model (Program R, “mgcv” package) rankings based on Akaike’s Information Criterion corrected for smaller sample sizes (AICc; hollow predictors versus guano mass response). A Gaussian family smoothing function was used with the “identity” link function (n = 131). CeilingHeight = height from top of opening to ceiling; MaxWidth = maximum width of hollow opening; Volume = calculated cone shape inside of hollow; MaxHeight = maximum height of hollow opening; DBH = Diameter at Breast Height.

Model	df	logLik	AICc	Delta AICc	Weight
CeilingHeight +MaxWidth	7	−73.93	152.23	0.00	0.22
CeilingHeight +Volume	7	−73.48	152.30	0.07	0.21
CeilingHeight +MaxHeight	7	−74.86	152.33	0.10	0.21
CeilingHeight	5	−74.82	152.55	0.32	0.19
CeilingHeight +MaxWidth +Volume	9	−73.19	153.82	1.59	0.10
CeilingHeight +DBH	7	−74.56	154.31	2.08	0.08
Null Model	1	−81.43	169.05	16.82	-

## Data Availability

Data supporting reported results is publicly available at Cal Poly Humboldt Digital Commons: https://digitalcommons.humboldt.edu/data/5.

## Supplementary Materials

The following supporting information can be downloaded at: https://www.mdpi.com/article/10.3390/ani12212950/s1, Figure S1: Study sites with search areas and hollow locations; Figure S2: The Generalized Additive Mixed Model associating the top variable ceiling height with guano mass.
