# Supplementary material for: Bat Use of Hollows in California’s Old-Growth Redwood Forests: From DNA to Ecology
Source: Animals (Basel). 2022 Oct 27;12(21):2950. doi: 10.3390/ani12212950 (PMC9656598; doi:10.3390/ani12212950)

animals

Article

Bat Use of Hollows in California's Old-Growth Redwood Forests: From DNA to Ecology

Amon J. Armstrong, Faith M. Walker, Colin J. Sobek, Cheri J. Sanville, Stephanie L. Martin and Joseph M. Szewczak

Figure S1. Study sites with search areas (purple) and hollow locations (yellow circles; sized by guano mass).

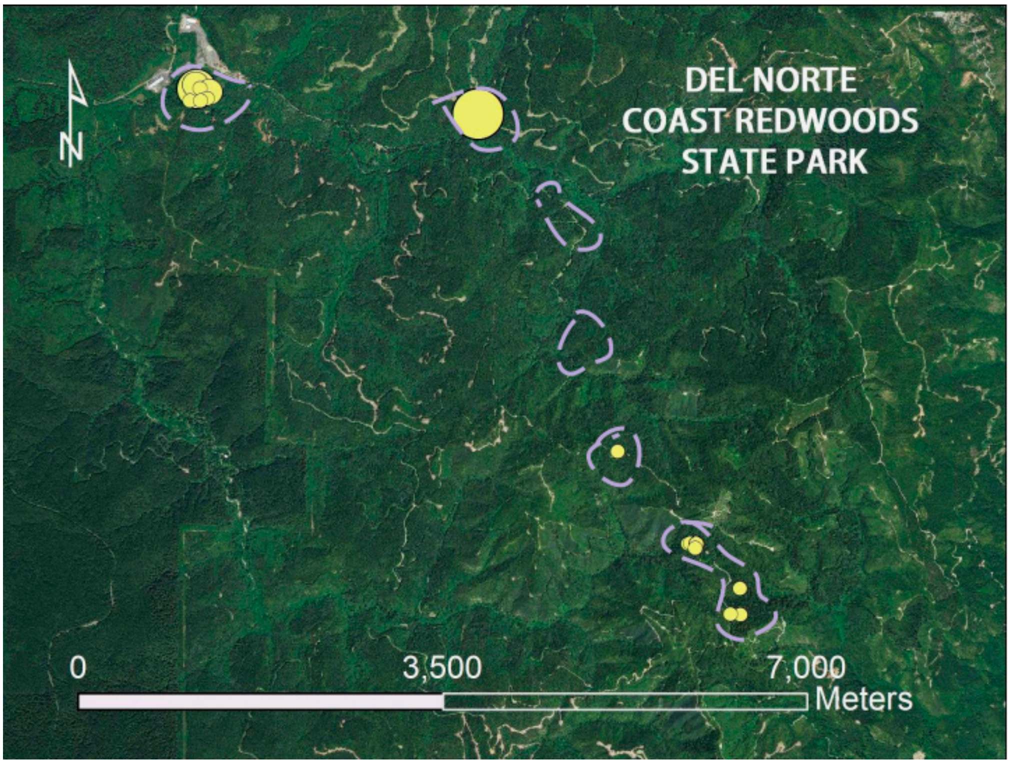

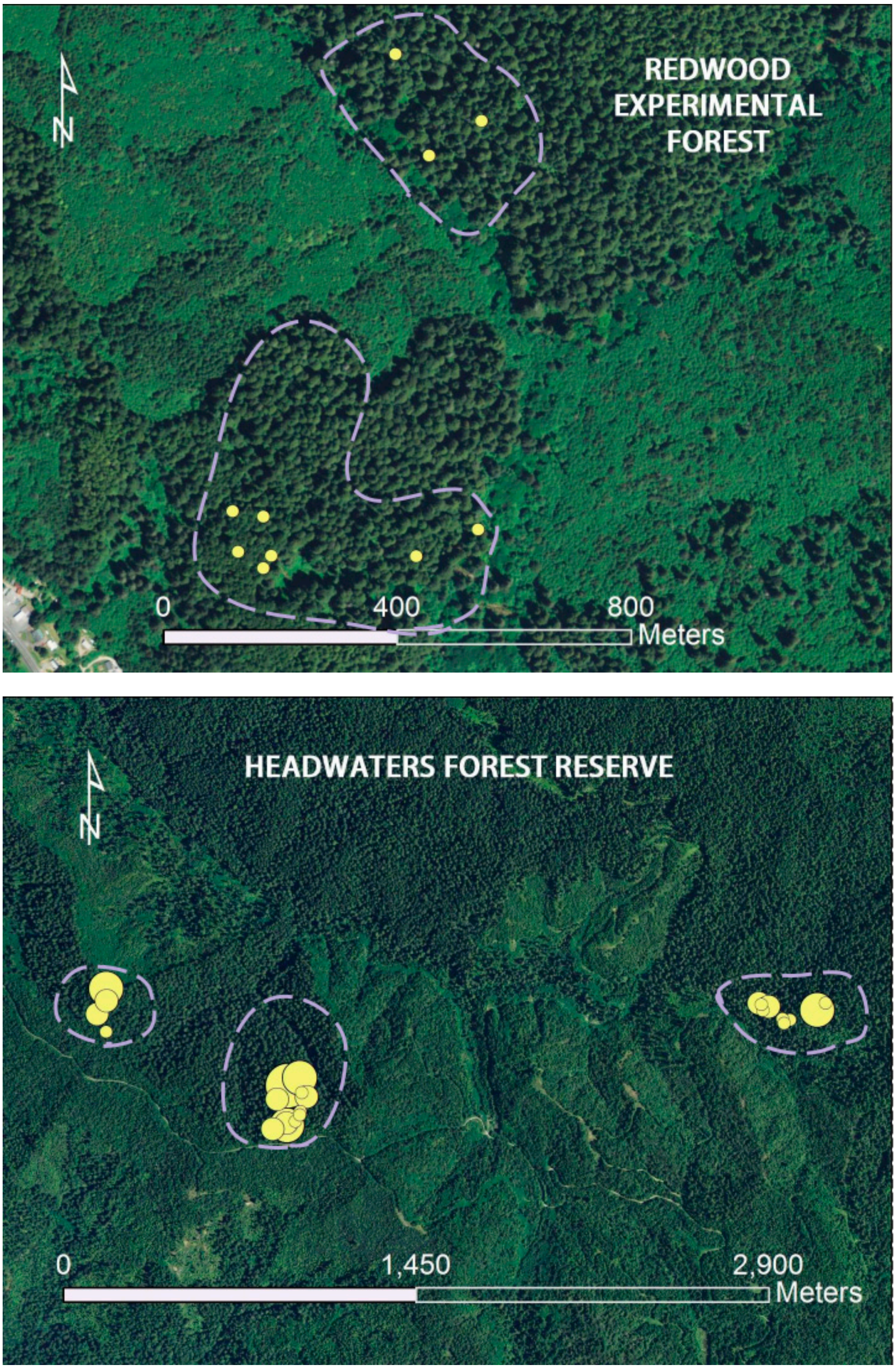
N
REDWOOD
EXPERIMENTAL
FOREST
0
400
800
Meters
N
HEADWATERS FOREST RESERVE
0
1,450
2,900
Meters

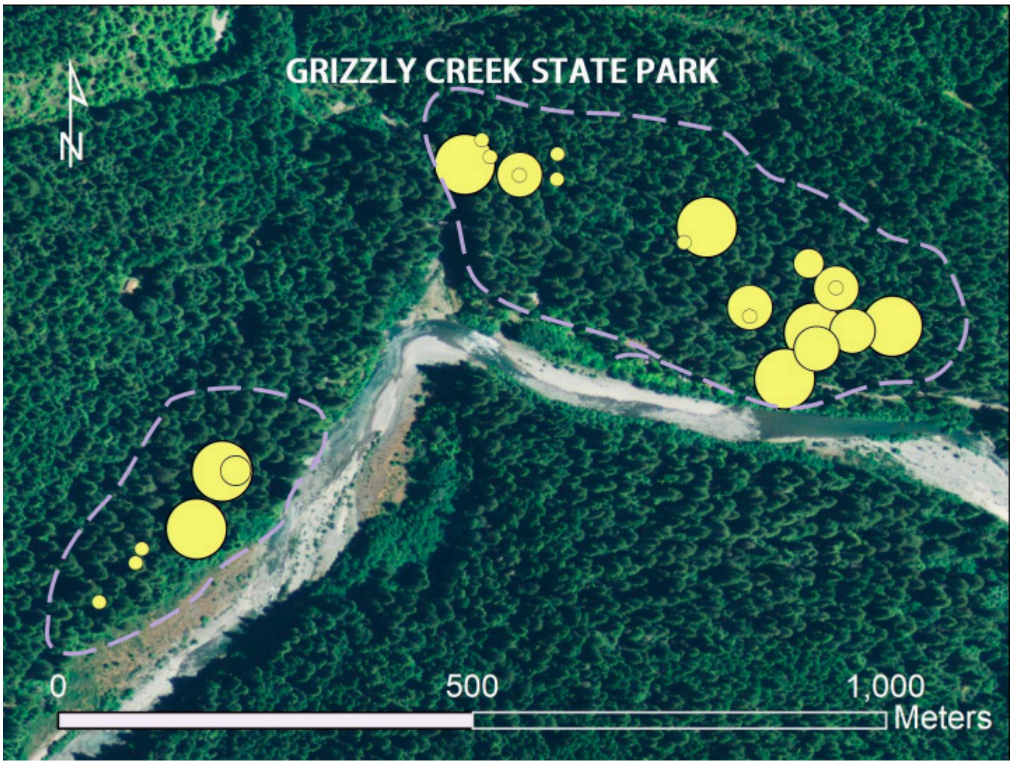
GRIZZLY CREEK STATE PARK
N
0
500
1,000
Meters

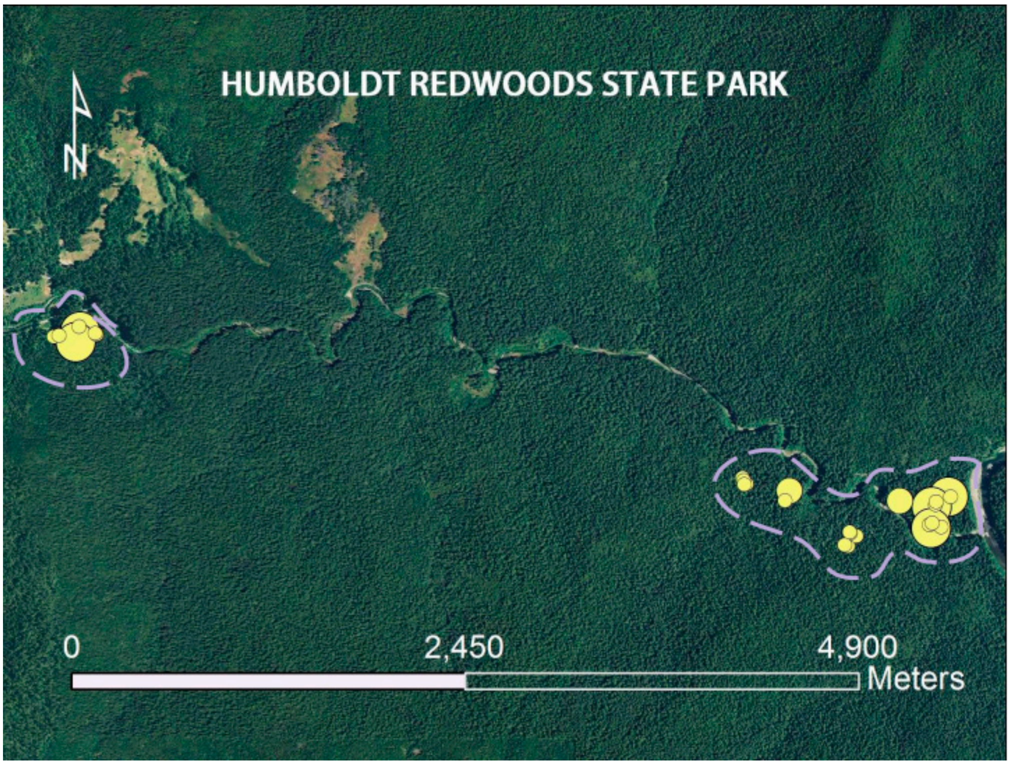
HUMBOLDT REDWOODS STATE PARK
N
0
2,450
4,900
Meters

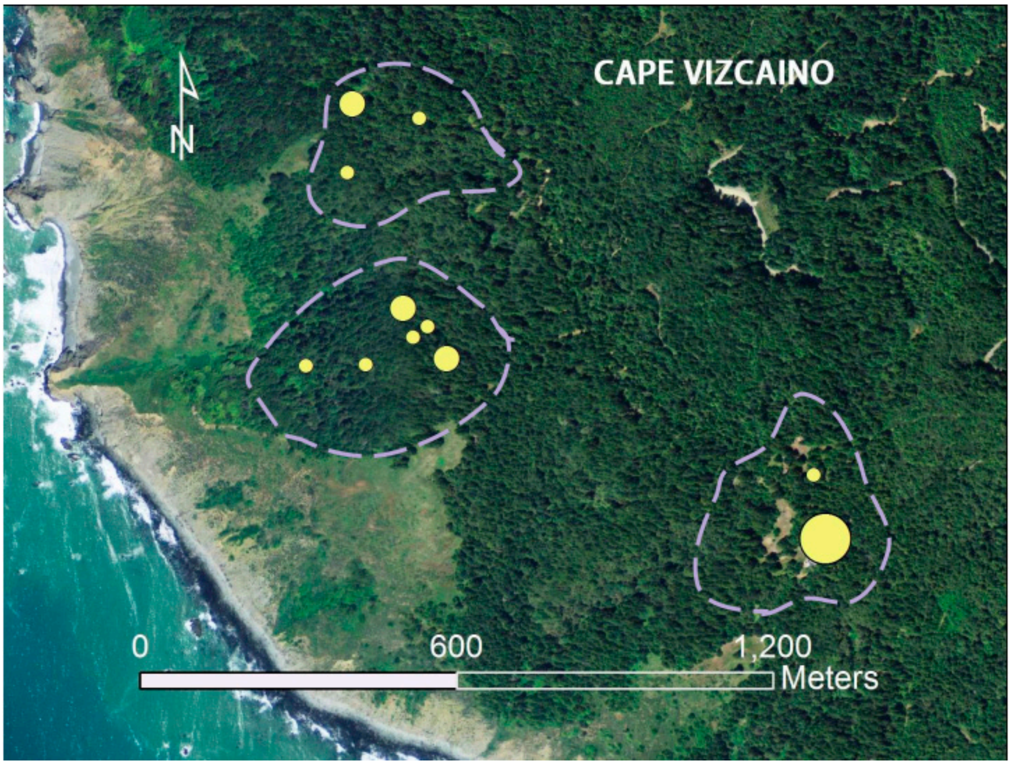
CAPE VIZCAINO
N
0
600
1,200
Meters

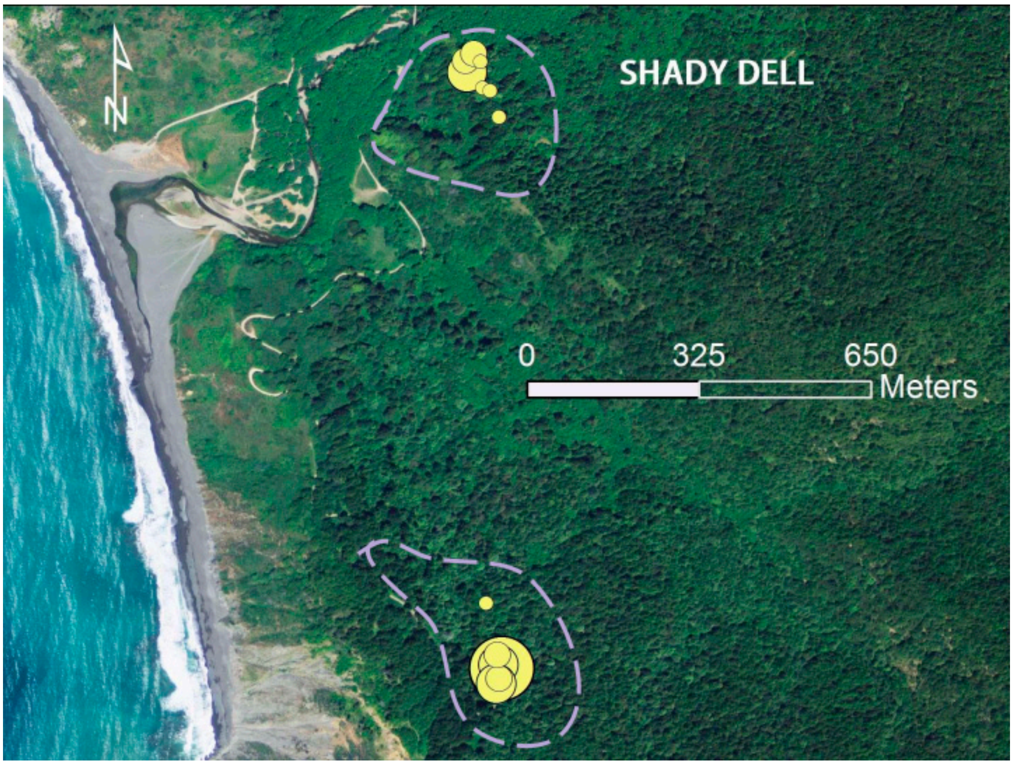
SHADY DELL
N
0
325
650
Meters

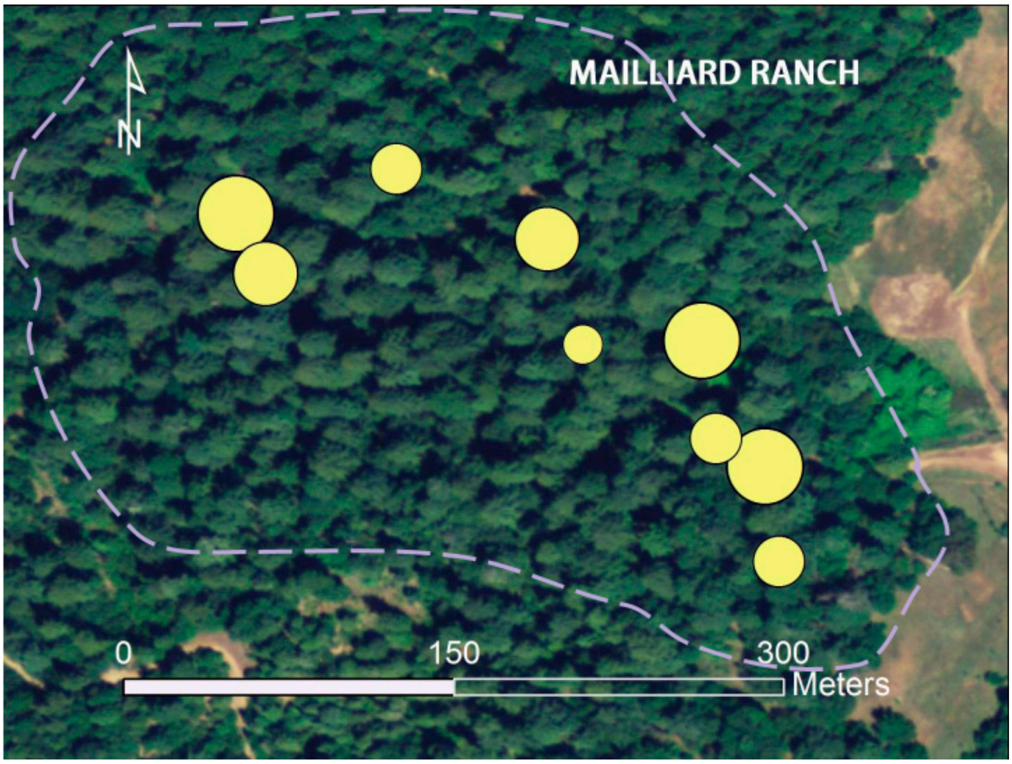

Figure S2. The Generalized Additive Mixed Model (GAMM; blue line) associating the top variable ceiling height with guano mass (adjusted R^2 = 0.17). Shaded areas are Bayesian posterior standard error fits (similar to 95% confidence intervals; "mgcv" package; Wood, S. N. 2020).

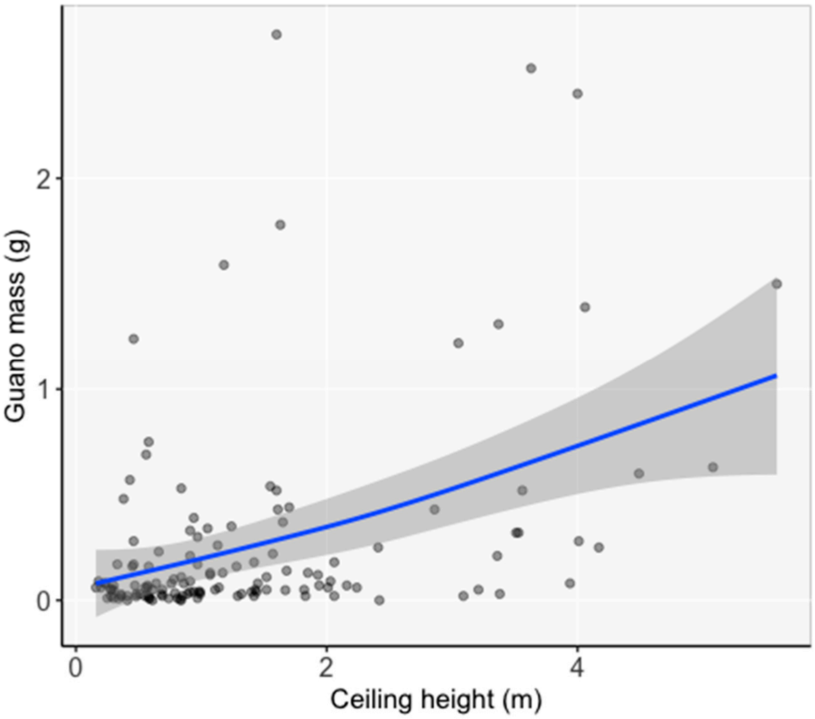

Supplement: Supplementary file 1 [file animals-12-02950-s001.zip › animals-1975403-supplementary.pdf]
